# Effectiveness of the Conservative Surgical Management of the Ameloblastomas: A Cross-Sectional Study

**DOI:** 10.3389/froh.2021.737424

**Published:** 2021-10-29

**Authors:** André Caroli Rocha, Felipe Paiva Fonseca, Alan Roger Santos-Silva, Silvia Vanessa Lourenço, Marcelo Minharro Ceccheti, Jayro Guimarães Júnior

**Affiliations:** ^1^Oral and Maxillofacial Surgery and Traumatology Service, Medical School, Clinical Hospital, University of São Paulo (FMUSP), São Paulo, Brazil; ^2^Oral Diagnosis Department, Piracicaba Dental School, University of Campinas (UNICAMP), Campinas, Brazil; ^3^General Pathology Department, Dental School, University of São Paulo, São Paulo, Brazil

**Keywords:** ameloblastoma, odontogenic tumors, treatment, curettage, cryotherapy

## Abstract

Ameloblastoma is a benign, but locally aggressive odontogenic neoplasm, whose appropriate therapeutic management remains highly debatable. The aim of this study was to evaluate the reliability and effectiveness of the two conservative surgical therapeutic protocols (curettage with peripheral ostectomy only and curettage plus cryotherapy) for the management of ameloblastomas. About 53 cases of the ameloblastomas treated in 9 years were retrospectively analyzed regarding their clinical, histopathologic, radiographic, and therapeutic data. The results and the postoperative complications related to both the therapeutic protocols were also statistically investigated. A slight female preponderance was seen (1.12:1.0) with a mean age of 27.1 years. The posterior mandible was the most affected site and dental involvement was frequently found. Multilocular lesions causing the alterations of the bone cortices were the most common radiographic findings. Recurrences were seen in 9.4% of the cases and although the patients submitted to curettage plus cryotherapy have shown an increased incidence of wound dehiscence, infection, and paresthesia, only bone sequestration proved to be significantly more frequent in this group compared to the patients treated by curettage with peripheral ostectomy only. The incidence of the recurrences following the conservative management is low and cryotherapy use as an adjuvant tool must be rationally considered.

## Introduction

Ameloblastoma represents the most or the second most common odontogenic tumor depending on the source of the sample analyzed and the criteria used [1–3]. This well-known benign neoplasia is clinically characterized by a local invasive and destructive potential odontogenic tumor, with a higher level of recurrences, if compared to the other odontogenic tumors. In the WHO classification (2017), this entity is divided into the conventional ameloblastoma, unicystic type, and peripheral ameloblastomas [4]. Slight gender predilections are variably described, but with no significant differences, whereas conventional ameloblastomas are more often diagnosed in the older individuals if compared to the younger patients affected by their unicystic counterparts [5].

Radiographically, ameloblastomas might reveal well-defined unilocular images or multilocular bone destructions, frequently causing tooth displacement or root resorption. Because the unicystic ameloblastomas are believed to behave less aggressively, a more conservative surgical approach is frequently chosen for these patients; however, due to the higher levels of the local recurrences associated with this therapeutic modality, segmental resections with the safety margins associated or not with the adjuvant approaches still represent the most common treatment for all the types of the ameloblastomas [6–8].

Nevertheless, the aggressive interventions are frequently associated with several local complications such as the impossibility of undertaking a satisfactory reconstruction because of the great amount of bone loss and the presence of the esthetic and functional impairments, easily illustrating the high degree of morbidity associated with these techniques. On the other hand, the reliability of the conservative approaches for treating the patients affected by the ameloblastomas is still being intensely investigated due to the high recurrence rate associated with this modality. However, it remains highly debatable if local recurrence would necessarily denote an unsuccessful approach considering the morbidity and quality of life of the patients, showing that the recurrence rate is not always the primary factor to consider in the treatment of ameloblastomas.

Hence, the aim of this study was to describe the clinicopathological and radiographic features of 53 cases of the ameloblastomas and to analyze the effectiveness of the two conservative therapeutic modalities (curettage only and curettage plus cryotherapy) in the management of this tumor.

## Materials and Methods

### Patients Enrolled

In 9 years from 1997 to 2006, the clinicopathological and therapeutic data of the patients affected by the ameloblastomas and surgically treated by the same surgeon (ACR), who has 25 years of experience and is a reference for the treatment of bone pathology in Brazil and Latin American countries, at the Department of Oral and Maxillofacial Surgery of the Clinical Hospital of University of São Paulo Medical School and followed at least for 2 years, were retrospectively reviewed from their medical charts.

Panoramic radiographs of the patients were retrieved and the tumor characteristics, including uni or multilocularity, tooth displacement or involvement, root resorption, and lesion size, were investigated. Analysis of the cortical involvement or disruption was done by evaluating a CT scan. H&E-stained histologic sections of all the cases were reviewed by an oral pathologist to confirm the original diagnoses and to further classify the cases according to their microscopic subtypes following the WHO guidelines.

About 73 patients were initially enrolled in the study; however, 10 patients did not comply with the minimum 2 years time of follow-up, one patient did not undergo treatment, one patient died due to an unrelated cause, one patient had the diagnosis changed after re-evaluation of the histologic sections, and seven patients were treated by another surgeon, being such cases excluded from the studied sample; therefore, remaining 53 cases of the patients had to be analyzed.

### Surgical Technique for the Conservative Treatment

Following the protocol of the institution, the curettage and curettage with cryotherapy are the two options for a conservative approach. Therefore, only curettage was performed in the cases that presented the large and irregular cavities with the tiny bone remnants, but in the well-circumscribed ameloblastomas, minimally affecting the cortical bone and presenting sufficiently thick bone, curettage plus cryotherapy was the option for the treatment. However, cases that presented a large extension with unclear margins and compromised the entire cortical bone without sufficient remnant or ineffective conservative treatment were treated with surgical resection. In all the cases where the conservative approaches were performed, intraoral access was used. The oral mucosa adjacent to the neoplasm or directly associated with it in cases of cortical rupture was removed. In addition, the surgical flaps of the periosteum were obtained and the teeth associated with the tumor were removed. Necessary ostectomy for the adequate access of the tumor was done so that the lesion could be removed as entirely as possible. Following macroscopic removal of the tumor, a vigorous curettage of the surgical field was performed with the purpose of removing all the peritumoral bone and the peripheral ostectomy was also done for correcting the osseous irregularities, especially in the multilocular lesions. A cryosurgery device containing liquid nitrogen (CRY-AC® Brymill Cryogenic Systems, Ellington, Connecticut, USA) was used in the three freeze/thaw cycles for the cases treated with the adjunct cryotherapy by using the visual aspect as a local parameter for bone freezing. Primary closure of the surgical field by using the adjacent oral mucosa was attempted in all the cases.

### Statistics and Ethical Statement

Statistical analyses were done by using the *t*-test and the ANOVA test for the numerical variables and the Fisher's exact test and the chi-squared test for the qualitative variables. The Kaplan–Meier curve was obtained to evaluate the disease-free survival of the patients. The Minitab Statistical Software version 15.0, State College, Pennsylvania, USA was used and a *p* < 0.05 was considered to be statistically significant.

This study was approved by the Research Ethics Committee of the University of São Paulo (Protocol 35/07) following the Declaration of Helsinki on the basis of the medical protocol and ethics. All the participants signed an informed consent agreement.

## Results

The clinicopathological, radiographic, and therapeutic data are summarized in Table 1. A slight female preponderance (1.12:1.0) and a mean age of 27.1 years (range 8–64 years) were found in the sample studied. An asymptomatic swelling represented the most common complaint (73.9%) with a mean duration time of 12.7 months (±22.5 months; range 1–120 months). The posterior region of the mandible was the most affected in location (40 cases or 75.5%), whereas the maxilla was involved in only three cases, always in the posterior area. One case proved to be confined to the soft tissue of a patient previously submitted to the resection of the tumor.

**Table 1:** Clinicopathological, radiographic, and therapeutic features of the patients affected by the ameloblastoma.

**Clinicopathological, radiographic, and treatment features**		**No**.	***%***
Sex	Male	25	47.2
	Female	28	52.8
Mean age	27.1 years (±13.8 years)		
Location	Posterior mandible	40	75.5
	Anterior mandible	4	7.6
	Anterior and posterior mandible	5	9.4
	Maxilla	3	5.7
	Soft tissue^*^	1	1.9
Clinic presentation	Swelling	34	64.2
	Swelling + pain	12	22.6
	Radiographic finding	7	13.2
Radiographic presentation	Unilocular	17	32.7
	Multilocular	35	67.3
Cortical involvement	**Vestibular/lingual cortices (*****n*** **=** **52)**		
	Expanded	5	9.6
	Perforated	1	1.9
	Expanded + Perforated	44	84.6
	Preserved	2	3.8
	**Basal cortical (*****n*** **=** **49)**		
	Expanded	25	51
	Perforated	0	0.0
	Expanded + Perforated	3	6.1
	Preserved	21	42.8
Dental involvement	**Tooth displacement**		
	Yes	39	73.6
	No	14	26.4
	**Root resorption**		
	Yes	36	67.9
	No	17	32.1
	**Unerupted tooth**		
	Yes	20	37.7
	No	33	62.3
Microscopic findings	Follicular	17	32.1
	Plexiform	17	32.1
	Mixed	11	20.8
	Basal cell	1	1.9
	Acanthomatous	1	1.9
	Unicystic	6	11.3
Previous treatment	Yes	17	32.1
	No	36	67.9
Treatment modality	Curettage	18	33.9
	Curettage + Cryotherapy	30	56.6
	Mandibulectomy	4	7.6
	Soft tissue resection	1	1.9

* *In this case, the lesion was located in the soft tissue of a patient previously submitted to the resection of the tumor*.

From the 52 central cases analyzed, 67.3% (35 cases) proved to be multilocular lesions, whereas 32.7% (17 cases) revealed a unilocular radiographic appearance. Vestibular and lingual osseous plates were frequently altered, being preserved in only two cases (3.8%), whereas basal cortical plates were uninvolved in 21 cases (42.8%). Considering the dental involvement, 39 patients presented tooth displacement, 36 patients showed root resorption, and 20 patients revealed unerupted teeth. The size of the lesions ranged in their greatest diameter from 5 to 115 mm with a mean size of 62 mm (±25.8 mm). Finally, the younger patients presented significantly more unilocular appearance compared to the older individuals, who more frequently revealed multilocular lesions (*p* < 0.006).

Microscopically, the follicular and plexiform subtypes predominated (32.1% each), whereas a mixed presentation was seen in 20.8% of the cases, acanthomatous was seen in 1.9% of the cases, and the basal cell was seen in another 1.9% of the cases. The diagnosis of the unicystic ameloblastomas was done in 11.3% of the sample after the clinicoradiographic and microscopic evaluation.

In this study, most of the patients had not been previously submitted to any kind of therapy (36 cases or 67.9%). Among those who had already undergone treatment, most of them received curettage (18.9%), whereas resection (3.8%), decompression (3.8%), and cryotherapy (5.7%) were applied less frequently. In this study, the conservative approaches were the most commonly used methods, where 56.6% of the patients were treated by curettage plus cryotherapy, 33.9% of the patients were treated by curettage with peripheral ostectomy only, 7.6% of the patients were treated by segmental mandibulectomy, and 1.9% of the patients were treated by soft-tissue resection. When the use of the conservative approaches was compared according to the frequency of the postoperative complications, bony sequestrum was significantly more present in the cases treated by curettage plus cryotherapy compared to the cases treated by curettage only (*p* = 0.017). On the other hand, no significant correlation was seen with the occurrence of the wound dehiscence, infection, transitional or permanent paresthesia, and pathologic fracture. Alterations in the facial contour were found in the three cases treated by curettage plus cryotherapy, in the two cases treated by curettage only, and in all the four cases treated by segmental mandibulectomy (Table 2).

**Table 2:** Frequency of the post-operative complications, time of follow-up, and incidence of the recurrences according to the therapeutic modality used.

	**Curettage (*n* = 18 cases)**	**Curettage + Cryotherapy (*n* = 30 cases)**	**Mandibulectomy (*n* = 4 cases)**	**Soft tissue resection (*n* = 1 case)**
Post-operatory side effect
Dehiscence	16 (88.9%)	26 (86.7%)	0 (0.0%)	0 (0.0%)
Infection	1 (5.6%)	5 (16.7%)	0 (0.0%)	0 (0.0%)
Transitory paresthesia	10 (55.6%)	17 (56.7%)	0 (0.0%)	0 (0.0%)
Permanent paresthesia	4 (22.2%)	10 (33.3%)	4 (100%)	0 (0.0%)
Pathologic fracture	0 (0.0%)	6 (20%)	0 (0.0%)	0 (0.0%)
Bone sequestrum^*^	1 (5.6%)	12 (40%)	0 (0.0%)	0 (0.0%)
Facial asymmetry	2 (11.1%)	3 (10%)	4 (100%)	1 (100%)
Time of follow-up^†^	55.5 (±26.8)	69.7 (±27.6)	74.2 (±34.2)	104.8
Recurrences	1 (5.6%)	3 (10%)	0 (0.0%)	1 (100%)

* *Statistically significant difference between the curettage and curettage plus cryotherapy (p = 0.017)*;

† *months*.

**Figure 1:**
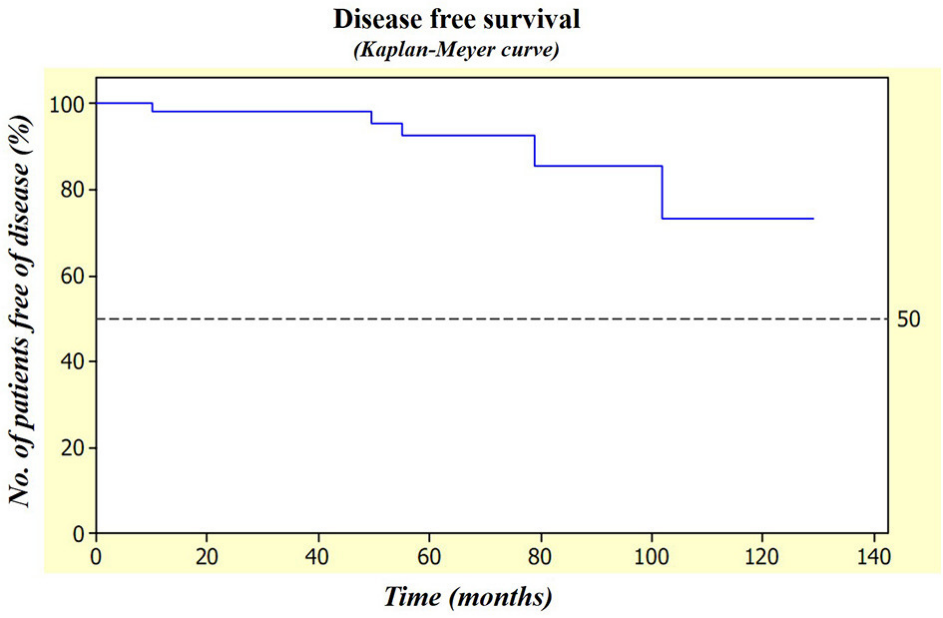
Patients in this study revealed a high disease-free survival rate measured by the Kaplan–Meier curve reaching almost 90% after 5 years of follow-up.

Clinical follow-up ranged from 24.4 to 128.9 months (mean of 65.8 ± 28.4 months) and in this period, recurrences were seen in the five cases (9.4%), being diagnosed from 10.1 to 101.8 months after surgery. In the four cases, the tumor was in the posterior region of the mandible, whereas in one case, neoplasia was found in the soft tissue adjacent to the previous resection margin. Three recurrences were seen in the patients treated by curettage plus cryotherapy, one recurrence was seen in a patient treated by curettage, and one recurrence was seen in a patient treated by soft-tissue resection. Among these five patients, three patients had received the previous treatment elsewhere. Additionally, we observed that the three cases with recurrence after curettage plus cryotherapy were found in the patients ≤ 30 years old, one was a male and two were females. They all presented the ameloblastomas measuring ≤ 50 mm with multilocular radiographic appearance causing dental dislocation, root resorption, and cortical perforation. In addition, the three cases presented wound dehiscence and one case of them also had bone sequestration after the treatment. However, no significant association was obtained between the recurrence and therapeutic approach, age, tumor location, radiographic features, microscopic subtype, or previous treatment, but a significant correlation was obtained between the recurrences and tumors with the smaller sizes (*p* = 0.011). It is essential to highlight that in most cases of recurrence, the time of follow-up was not as rigorous as planned due to the uncontrollable reasons inherent to the patients. Nevertheless, all the patients are currently alive, one of them with the disease. Figure 1 illustrates the disease-free survival rate of the sample studied.

## Discussion

Ameloblastoma is considered the odontogenic tumor with major clinical significance, especially because of its prevalence, clinical behavior, higher incidence of recurrences, and therapeutic controversies. The tumor normally reveals a slow infiltrative growth pattern that may cause not only the local swellings but also large facial asymmetries. Pain is usually absent and may delay its initial diagnosis. The clinical features observed in this study confirm these characteristics, once over 83% of the patients chiefly complained of an asymptomatic swelling with a mean time of evolution of more than 1 year, which is in conformity with the previous descriptions that also found the durations longer than 1 year before the diagnosis can be performed [6–9].

In this study, the posterior region of the mandible is by far the most affected location and no significant difference can be seen between the men and women in most of the series, although a slight female preponderance is occasionally found [6, 10]. Patients affected by the ameloblastomas usually reveal a broad age range with a peak incidence in the 3rd and 4th decades of life [11]. This data is in accordance to be described by Reichart et al. [12] for the patients from the developing countries and Bataineh [13] and Vayvada et al. [14] suggesting that a delay in the early diagnostic process would be responsible for the differences in the mean age of the patients affected by the ameloblastomas in the different regions of the globe [5].

Radiographically, ameloblastomas are usually found as uni or multilocular radiolucid defects that frequently cause cortical disruptions, dental displacement, or root resorptions [13, 15]. Because the unicystic ameloblastomas are more commonly diagnosed in the younger patients, the higher frequency of unilocular images significantly found in the younger patients was not unexpected and it is similar to the results reported by the other authors [16, 17]. These cases are believed to behave less aggressively, which is consistent with the findings that all the four central recurrent cases were multilocular lesions that caused the cortical perforations and involvement of the basal plate. Since the dental involvement represents a common radiographic finding, we observed a higher incidence compared to the described above. Ogunzalu et al. [18] observed 36% of the tooth involvement against 73.6% in the current survey, whereas Poo et al. [19] described 11.9% of the unerupted teeth associated with the tumor. Siar et al. [8] reported 6.7% of the root resorptions, whereas we found 37.7% and 67.9% of the root resorptions, respectively.

The size of the tumors has only rarely been evaluated and described. A study by Sampson et al. [20] investigates that the mean size of 62 mm is higher compared to the mean size of 50 mm, but it also showed that the smaller tumors significantly correlated with the recurrences, suggesting that the factors other than the tumor size might be important for predicting the treatment failure and confirming that the locally infiltrative lesions may be more difficult to treat compared to the extensive, but well-circumscribed neoplasias. In this study, all the central recurrent lesions presented the cortical perforations, even though they revealed smaller diameters compared to those not recurred (47 vs. 63.6 mm), further suggesting that the growth pattern with the cortical disruptions might be more indicative of the recurrence compared to the neoplastic linear dimensions.

In this study, the histologic variants of ameloblastoma are well-recognized and their lack of association with the clinical features is well-accepted and the follicular and plexiform subtypes are by far the most common [10, 15, 21]. However, their mixed presentation usually overcomes their frequency, especially when the surgical specimens are evaluated, instead of the small biopsy materials. On the other hand, despite the importance of recognizing unicystic presentation of the ameloblastomas because of their already mentioned less aggressive behavior, definitive unicystic diagnosis is difficult to be accomplished and it is often accepted only after the macroscopic and histologic analyses of the surgical specimens, making its distinction from the conventional ameloblastomas a useless approach [10]. The incidence of unicystic ameloblastomas normally ranges from 5 to 20% of all the cases diagnosed, more frequently between the 2nd and 3rd decades of life [11]. Hence, the 11.3% incidence observed in this cohort is in agreement with the literature, but the mean age of 15 years seems to be inferior [22, 23].

The best therapeutic modality still represents the most controversial topic about ameloblastomas. Two main groups can be currently discernible, the first one who supports the use of the more aggressive approaches, proposing the *en bloc* resection of the tumor with safety margins, and a second one that supports a more conservative approach, preserving the surrounding clinically non-involved bone that may or may not be followed by using an adjunct technique. The number of recurrences observed after the use of the first proposal is usually inferior, but it also exhibits an increased degree of morbidity and frequently requires the additional reconstructive procedures, whereas, despite the higher number of recurrences of conservative approaches, it represents a much less invasive modality and its use in the treatment of the ameloblastomas has gained more adoption in the last years, where 90.6% of the patients were treated by the conservative approaches and because of such high frequency of use, this study investigates the reliability and usefulness of these specific modalities [6, 11].

Cryotherapy is frequently used as an adjuvant technique in the conservative treatment of ameloblastomas because of its suggested potential for decreasing the number of recurrences by destroying the possible residual neoplastic foci after curettage [11]. However, as previously described in the literature, we also observed an important number of side effects related to the use of cryotherapies such as bony sequestrum, infection, pathological fractures, and paresthesias, suggesting that its use must be well-rationally indicated for each case. On the other hand, in both, groups treated conservatively, wound dehiscence was a frequent complication observed probably as a consequence of the lack of a supportive bone to stabilize the suture procedures infection and inflammation.

The use of invasive approaches in the treatment of infants is frequently contraindicated because of the possible disruptions in their facial growth potential, possibly leading to facial asymmetries [9]. In this study, 11 patients were below 16 years and, therefore, in the growth phase. All of them were treated conservatively and none revealed alterations in their facial development. Similarly, considering the whole sample conservatively treated, only a small index of facial asymmetry was observed in both the groups (11.1% curettage only and 10% curettage plus cryotherapy), which is in agreement with the literature and inferior to those found in the patients submitted to the resections [9].

Finally, the overall incidence of the recurrences observed in this study by using the conservative approaches was considered lower compared to described in the literature (5.6% for curettage and 10% for curettage plus cryotherapy) [23] that may be a consequence of a more cautious surgical procedure performed in one single institution that further eliminates the bias created by the different performances of the distinct surgeons, leading to an excellent disease-free survival of over 90% after 5 years. Moreover, the results of this study also support the use of the conservative approaches for the cases with involvement of the bone basal cortical, which has traditionally represented a contraindication for these therapeutic modalities. Taken together, the current results point out to the conservative curettage, even without adjuvant cryotherapy, as a valid methodology in the treatment of the ameloblastomas, with a small number of recurrences and acceptable postoperative complications that can be more easily managed (when promptly diagnosed and treated) compared to the experienced after the wide resections.

## Data Availability Statement

The raw data supporting the conclusions of this article will be made available by the authors, without undue reservation.

## Ethics Statement

The studies involving human participants were reviewed and approved by Research Ethical Committee of the University of São Paulo (Protocol 35/07). Written informed consent to participate in this study was provided by the participants' legal guardian/next of kin.

## Author Contributions

AR contributes to the design of the study and the surgical management of the patients. FF and AS-S contribute to the preparation of the manuscript. SL and MC contributes to the histopathological assessment of the surgical specimens, preparation of the manuscript, and surgical management of the patients. JJ contributes to the design of the study. All authors contributed to the article and approved the submitted version.

## Conflict of Interest

The authors declare that the research was conducted in the absence of any commercial or financial relationships that could be construed as a potential conflict of interest.

## Publisher's Note

All claims expressed in this article are solely those of the authors and do not necessarily represent those of their affiliated organizations, or those of the publisher, the editors and the reviewers. Any product that may be evaluated in this article, or claim that may be made by its manufacturer, is not guaranteed or endorsed by the publisher.
